# Bquant – Novel script for batch quantification of LCMS data

**DOI:** 10.1016/j.mex.2016.09.001

**Published:** 2016-09-14

**Authors:** Marko Rožman, Mira Petrović

**Affiliations:** a Catalan Institute for Water Research (ICRA), Carrer Emili Grahit 101, 17003 Girona, Spain; b Ruđer Bošković Institute, Bijenička cesta 54, 10000 Zagreb, Croatia

**Keywords:** Computer script for batch quantification of liquid chromatography mass spectrometry data, Liquid chromatography, Mass spectrometry, Automated routine, Quantification, Computer script, Data post processing, Emerging contaminants, Metabolites

## Abstract

Quantitative target analysis by liquid chromatography coupled to mass spectrometry (LCMS) is ubiquitous in environmental, metabolomic and toxicological studies. Targeted LCMS methods are capable of the simultaneous determination of literally hundreds of analytes. Although acquiring of instrumental data is very fast, data post-processing i.e. quantification can be time consuming step (and)or dependent to various commercial software packages. In attempt to facilitate this drawback Wolfram Mathematica script for batch quantification of LCMS data was created. Script works with direct outputs of integration algorithms created by different instrument control software’s or custom created outputs. Key benefits of Bquant script are:

• simple and automated routine for batch mode quantification
• vast improvement in processing time (especially compared to manual interpretation)
• data can be quickly re-analysed using different inputs

Script was validated on various datasets and some of these were provided as working examples.

## Graphical abstract

**Figure:**
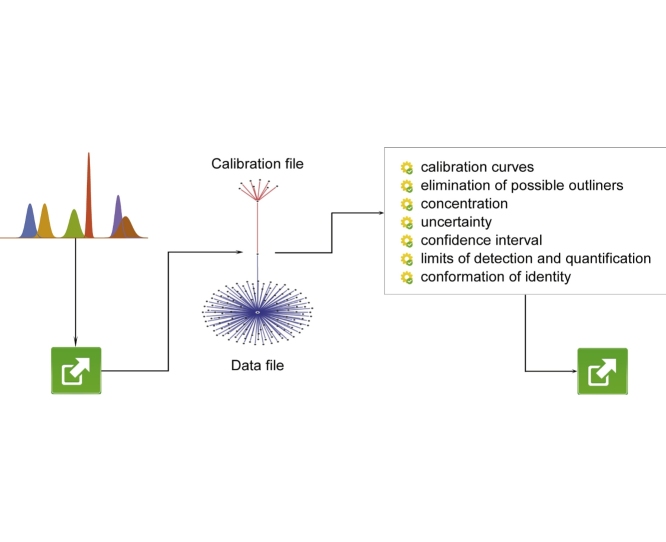

## Method

Here we present novel script for batch quantification of liquid chromatography mass spectrometry (LCMS) data. The script developed in this work was applied to quantify emerging contaminants in several European (FP7&H2020) and national projects e.g. Transformer, Globaqua and TransformCoast working on outputs of different instrument control software e.g. ThermoScientific Xcalibur, AB Sciex Analyst. In addition to environmental studies, the script can be applied for any quantitative analysis of different compounds in e.g. metabolomic or toxicological studies [1], [2]. This simple script enables quantification of data sets in a batch mode and shortens processing time from 20 min (manual interpretation) to several seconds per data set. Errors in data analysis are reduced and data can be quickly re-analysed using different inputs.

### Step 1: preparation of the data

Mass spectra processing is not performed by Bquant script. Component peak identification and integration have to be executed using instrument vendor software. All instrument control software performs these basic operations. Signals used for quantification (peak area) may represent the parent compound or more commonly the two most intense selected reaction monitoring transitions (in terms of peak area). Once integration is performed output should be saved as Microsoft Excel xlsx file(s). Outputs from Thermo Xcalibur Quan Browser, AB Sciex Analyst and custom prepared file are provided as an examples (can be downloaded as Supporting information). Two files should be generated: one containing data for the calibration curve (e.g. Calibration-Analyst.xlsx) and the other containing data of the analytes (e.g. Data-Analyst.xlsx). Of all parameters reported by integration, user must ensure that Excel files created contain columns and appropriate data listed in Table 1. In addition, input text file (input.txt) which contains instructions for the Bquant script should be provided. First row should include name of the output to be used i.e Analyst, Thermo or custom. Following five rows must contain (in this order): value of the square of the correlation coefficient e.g. 0.99, minimum number of points for constructing calibration curve, concentration of the internal standard, confidence interval e.g. 0.95 (for 95%) and True if *y*-intercept of the calibration curve is allowed (otherwise False). Input for analysis of Thermo Xcalibur data must include additional row with names of all compounds to be quantified in curly brackets e.g. {Azithromycin, Ciprofloxacin,…}.

### Step 2: running batch quantitative analysis

This step requires Wolfram Mathematica (script is tested for version 8 and higher, Wolfram Research, Oxfordshire, UK) proprietary mathematical computation program. Running the analysis on a modern computer (2 GB RAM) will suffice. Bquant script, test and result files can be downloaded as Supplementary material to this article. The latest public scripts and future developments like web-based tool are available at www.irb.hr/Bquant-download. In this section we demonstrate analysis of data provided in Analyst folder but all data follows the same pipeline. Workflow is very simple to perform since all steps represent scripted part.

Bquant script can be run directly from its location. Copy entire Bquant folder to desired location on your hard drive (e.g. D:\….\Bquant\). Choose folder and script on a basis of the instrument data (in this example …\Bquant\Analyst\Bquant-Analyst.nb). Open notebook file (nb extension) in Mathematica. Open Evaluation menu and go to Evaluate Notebook command. Before executing script make sure that Calibration, Data and input files are present and correctly formatted. Evaluate Notebook command will start script evaluation and batch processing of experimental data.

#### Script first reads the input parameters and loads the experimental data

**Fig. 1:**
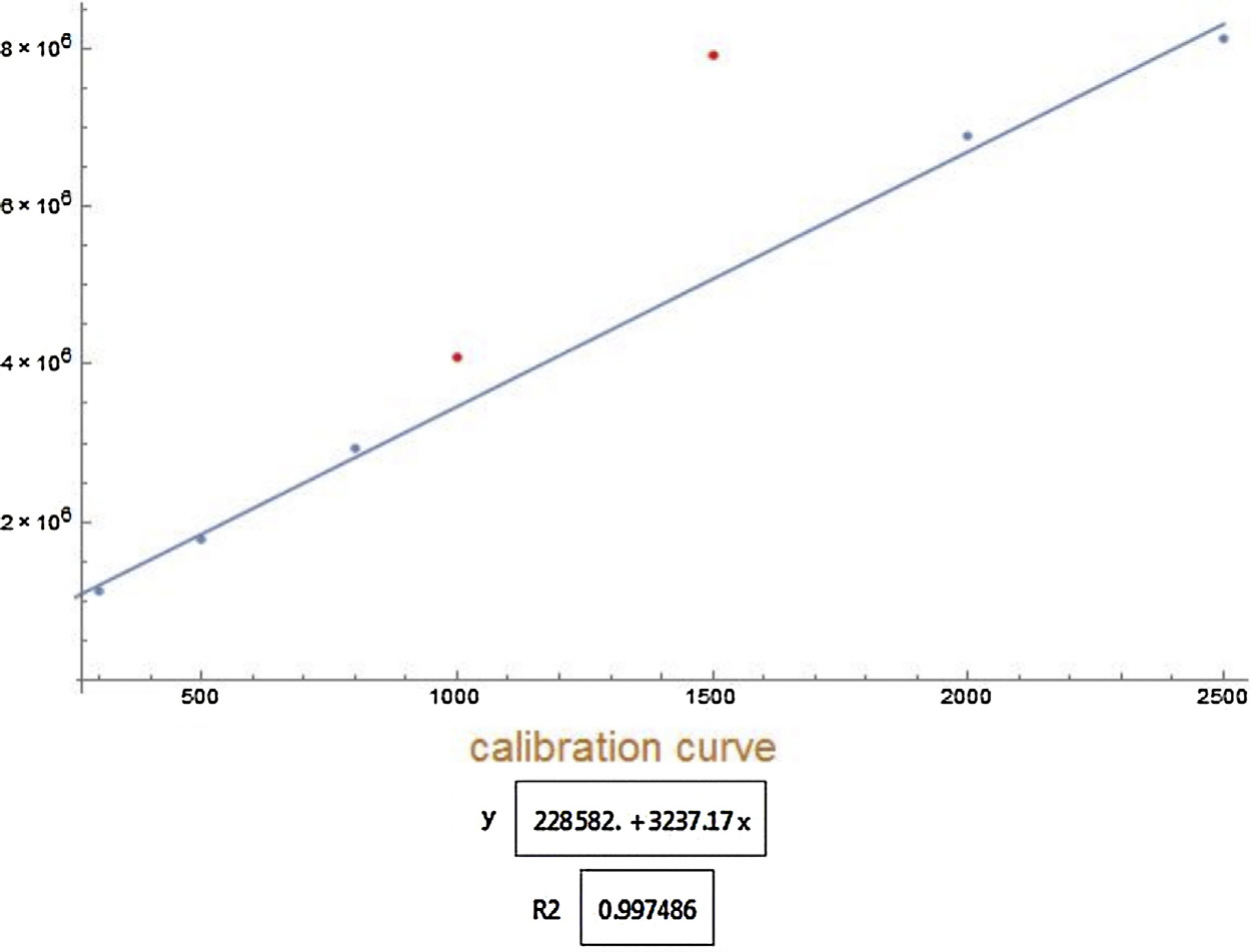
Example of the calibration curve. Blue dots represent data points used for generating curve while the outliers are depicted as red dots. Axes are not labelled due to possibility of using different units.

By using data from Calibration file a calibration curve that shows the response of an analytical method (analyte peak area) to known quantities of the analyte is constructed. Calibration curve is constructed using linear model. Square of the correlation coefficient, *R^2^* is used as measure of goodness of fit. In order to find best combination between number of data points and *R^2^* as well as to eliminate possible outliers subsets of data are formed. Subsets contain all combinations of data points ranging from minimal number predefined in input file and experimentally available data points. Linear model is evaluated for every subset and best combination is selected on the basis of *R^2^* value above defined limit (in input file) and maximal number of points. If such calibration curve cannot be constructed message is returned. Following instructions from input file *y*-intercept of the calibration curve can be forced to zero. Selected calibration curve with corresponding linear equation and *R^2^* value is provided in result file. It is advisable to always inspect the calibration curve. Example of constructed calibration curve is shown on Fig. 1.

In the next step functions for estimating uncertainty and confidence interval are defined. Uncertainty (±*u*) in analyte concentration (*x*) is represented by

$$\frac{\sigma_{y}}{\left|a\right|}\sqrt{\frac{1}{k}\;+\;\frac{1}{n}\;+\;\frac{{\left(y\;-\;\bar{y}\right)}^{2}}{a^{2}\;\Sigma \;{\left(x_{i}\;-\;\bar{x}\right)}^{2}}}$$

where *σ_y_* is the standard deviation of *y*, *a* is the value of the calibration curve slope, *k* is the number of replicate measurements, *n* is the number of data points for the calibration curve, *y* and *x* are values for the points on the calibration line [3], [4].

The confidence interval for *x* is ±*ut*, where *t* is Student’s *t* for (number of points −2) degrees of freedom [3], [4].

Limits of detection (LOD) and quantification (LOQ) are determined from the least-squares equation of a calibration curve [3], [4]. Quotient of standard deviation of *y* and slope of calibration curve is defined as measure of noise. LOD is set up to be three times greater than the noise while LOQ as ten times greater.

#### In the next step use of internal standard method is considered

Internal standard calibration curve, uncertainty, confidence interval, LOD and LOQ are calculated. In this method the response signal is not the analyte signal itself but the ratio of the area of analyte signal to the area of reference standard signal. Accordingly, concentration of the analyte is divided by concentration of internal standard. It is advisable to use the internal standard method [3], [4] as it can compensate for certain types of errors if these influence both the analyte and the internal standard e.g. sample injection, flow rate, column conditions, instrument response etc.

#### T1/T2 identity

For unequivocal identification and confirmation in typical quantification experiments two selected reaction transitions per compound are recorded. Usually two most abundant fragment ions were monitored, first quantifier and second qualifier. Following step calculates the intensity ratio between quantifier (first transition) and qualifier (second transition) using areas of first and second transition. Mean ratio is calculated for reference standards from Calibration file. Absolute deviation of every compound in the sample from established mean is reported. This serves as conformation of identity of compounds in the samples in addition to mass and LC retention time (used during integration process). Value and cut off limit for this parameter is left to the user and the result does not impede calculation of concentration. For compounds with just one transition reported or missing data for second transition “No Data” message is displayed.

#### Final step of the script creates result table with all properties calculated

Sample name, concentration, LOD, LOQ, uncertainty, confidence interval, concentration internal, LOD internal, LOQ internal, uncertainty internal, confidence interval internal, T1/T2 identity are reported. The result file (“name of the compound”-Results.xlsx) is saved in working directory. Examples of the result outputs can be found in supporting data e.g. in Anlyst folder Atorvastatin-Results.xlsx is example of full output, Ibuprofen-Results.xlsx is example of using just one transition and Hydrpchlorothiazide-Results.xlsx represents example when one calibration curve can’t be constructed within given parameters.

## Figures and Tables

**Table 1:** Format and content of the Excel files used by Bquant script.

Software	File name	Mandatory columns	Format
Analyst	Calibration-Analyst.xlsx	Analyte Concentration (ng/mL), Analyte Peak Area (counts), IS Peak Area (counts), Analyte Peak Name	Data should be provided in one Excel sheet. Analyte concentration does not have to be in ng/mL. If transitions are used they should be named as “name of the compound”-1 and “name of the compound”-2 under Analyte Peak Name column. Use the same compound name in Data file.
	Data-Analyst.xlsx	Sample Name, Analyte Peak Area (counts), IS Peak Area (counts), Analyte Peak Name	Use Sample Name for identifying the samples.
Xcalibur	Calibration-Xcalibur.xlsx	Sample ID, Area, ISTD Area	Batch quantification in Xcalibur saves every compound as different Excel sheet. If transitions are used make sure they are named as “name of the compound”-1 and “name of the compound”-2. Under sample ID provide concentrations of standards for calibration curve. Use the same compound name in Data file.
	Data-Xcalibur.xlsx	Sample ID, Area, ISTD Area	Here use sample ID for identifying the samples.
custom	Calibration-custom.xlsx	Analyte Concentration, Analyte Peak, IS Peak, Analyte Peak Name	Data should be provided in one Excel sheet. If transitions are used they should be named as “name of the compound”-1 and “name of the compound”-2 under Analyte Peak Name. Use the same compound name in Data file.
	Data-custom.xlsx	Sample Name, Analyte Peak, IS Peak, Analyte Peak Name	Use Sample Name for identifying the samples.

## References

[1] Schelli K., Rutowski J., Roubidoux J., Zhu J. (2016). Staphylococcus aureus methicillin resistance detected by HPLC-MS/MS targeted metabolic profiling. J. Chromatogr. B.

[2] Gajski G., Čimbora-Zovko T., Rak S., Rožman M., Osmak M., Garaj-Vrhovac V. (2014). Combined antitumor effects of bee venom and cisplatin on human cervical and laryngeal carcinoma cells and their drug resistant sublines. J. Appl. Toxicol..

[3] Harris D.C. (2010). Quantitative Chemical Analysis.

[4] Skoog D.A., West D.M., Holler F.J., Crouch S.R. (2014). Fundamentals of Analytical Chemistry.

